# The clinical and immunological characteristics of COVID‐19 patients with delayed SARS‐CoV‐2 virus clearance

**DOI:** 10.1002/iid3.999

**Published:** 2023-09-29

**Authors:** Jinsong Wang, Debao Li, Bo Tang, Xuemin Sun, Wenjiong Shi, Hao Li, Zhenhua Zhang, Yuzhang Wu, Yi Zhang, Qinghua Qiao

**Affiliations:** ^1^ Institute of Immunology, PLA Army Medical University Chongqing China; ^2^ Department of Immunology Medical College of Qingdao University Qingdao Shandong China; ^3^ Chongqing International Institute for Immunology Chongqing China; ^4^ Pingdingshan Medical District the 989th Hospital of the PLA Joint Logistic Support Force Pingdingshan Henan China; ^5^ Department of Radiology the 989th Hospital of the PLA Joint Logistic Support Force Luoyang Henan China; ^6^ School of Pharmacy and Bioengineering Chongqing University of Technology Chongqing China

**Keywords:** COVID‐19, delayed clearance, immunological features, PD‐1, SARS‐CoV‐2

## Abstract

**Background:**

The outbreak of coronavirus disease 2019 (COVID‐19) caused by severe acute respiratory syndrome coronavirus 2 (SARS‐CoV‐2) has posed a great threat to human health. Some severe COVID‐19 patients still carried detectable levels of SARS‐CoV‐2 even after prolonged intensive care unit treatment. However, the immunological features of these COVID‐19 patients with delayed virus clearance (CDVC) are still unclear.

**Methods:**

We retrospectively reviewed the clinical and immunological data of 13 CDVC cases, who were admitted into one hospital in Wuhan from February to April 2020. These data were also compared to those of perished (*n* = 9) and recovered (*n* = 52) cases. The expression of the exhaustion marker PD‐1 on circulating T cells of these patients was measured by flow cytometry.

**Results:**

High levels of serum interleukin‐6 (IL‐6), IL‐1β, IL‐8, as well as other inflammatory mediators, were seen in CDVC cases. Severe lymphopenia was observed in CDVC patients with the counts of total lymphocytes (0.9 × 10^9^/L), CD4^+^ T cells (0.35 × 10^9^/L), and CD8^+^ T cells (0.28 × 10^9^/L) below their corresponding lower limits of normal range. Similar to the perished group, CDVC cases have higher percentages of CD25^+^Foxp3^+^ regulatory T cells (Treg) in circulation. Moreover, enhanced expression of the exhaustion marker PD‐1 on CCR7^−^CD45RA^+^ effector, CCR7^+^CD45RA^−^ central memory, and CCR7^−^CD45RA^−^ effector memory CD4^+^ and CD8^+^ T cells were also observed in CDVC cases.

**Conclusion:**

CDVC patients still have SARS‐CoV‐2 and these cases manifest with severe clinical symptoms due to persistent inflammation. Augmentation of the frequency of circulating Treg, severe lymphopenia, and functional exhaustion of T cells might lead to inefficient clearance of SARS‐CoV‐2. Therefore, enhancing lymphocyte counts and reversing T‐cell exhaustion might be key methods to boost immune responses and eliminate SARS‐CoV‐2 in CDVC patients.

## Graphical abstract

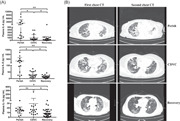

## 1 INTRODUCTION

In December 2019, cases of pneumonia of unknown etiology were reported in Wuhan, Hubei Province, China. Later, the causative agent of this severe pneumonia was identified as a novel coronavirus and named as 2019 novel coronavirus (2019‐nCoV), and subsequently officially reclassified as the novel severe acute respiratory syndrome coronavirus 2 (SARS‐CoV‐2), while the clinical condition is referred to as coronavirus disease 2019 (COVID‐19) by the World Health Organization (WHO).^1^, ^2^, ^3^, ^4^ The number of infected cases and COVID‐19‐associated deaths are still increasing daily.

The typical symptoms of COVID‐19 usually appear 2–14 days after viral exposure. Fever, cough, shortness of breath, and pneumonia are very common in mild cases, whereas severe cases show respiratory, hepatic, gastrointestinal, and neurological complications.^5^, ^6^ Lymphopenia and “cytokine storms” are also prevalent in COVID‐19 patients, especially in aged and critically ill cases, thus leading to intensive care unit (ICU) admission and high mortality.^7^, ^8^, ^9^ At the end of January 2020, the Chinese government decided to set up the Huoshenshan Hospital in Wuhan to treat patients with severe and critically ill cases of COVID‐19. After 10 days of construction (from January 24 to February 4, 2020), this hospital was successfully built and began to admit COVID‐19 patients. Over 60 days of continuous treatment, these COVID‐19 patients have had three outcomes: a small proportion of cases perished, most cases were recovered, and some patients were persistently tested SARS‐CoV‐2 positive by use of quantitative RT‐PCR (qRT‐PCR) of throat swab samples. We here term these cases as COVID‐19 patients with delayed virus clearance (CDVC). However, information on immunological features of CDVC patients is still unclear.

In this study, we performed a comprehensive evaluation of clinical and immunological data of 74 patients admitted to Huoshenshan Hospital, Wuhan. These patients were categorized into three groups including perished (*n* = 9), CDVC (*n* = 13), and recovered (n = 52) based on outcomes of treatment. Our findings may extend the knowledge and understanding of the factors associated with CDVC.

## 2 METHODS

### 2.1 Patients

Electronic medical records from 74 patients with confirmed COVID‐19 and admitted into the first area of pneumonia in the Huoshenshan Hospital from February 5th to April 10th, 2020, were collected and retrospectively analyzed. Diagnosis of COVID‐19 was based on the New Coronavirus Pneumonia Prevention and Control Program (sixth edition) published by the National Health Commission of China. All the patients were laboratory‐confirmed positive for SARS‐CoV‐2 by use of quantitative RT‐PCR (qRT‐PCR) of throat swab samples. Primers and probes targeting the SARS‐CoV‐2 envelope gene were used and the sequences were as follows: forward primer, 5′‐TCAGAATGCCAATCTCCCCAAC‐3′; reverse primer, 5′‐AAAGGTCCACCCGATA CATTGA‐3′; and the probe 5′‐CY5‐CTAGTTACACTAGCCATCCTTACTGC‐3′‐BHQ1. Conditions for the amplifications were 50°C for 15 min, 95°C for 3 min, followed by 45 cycles of 95°C for 15 s and 60°C for 30 s.

### 2.2 Definitions

Based on the criteria outlined in the COVID‐19 Diagnosis and Treatment Plan (sixth edition), all patients included in this study were classified as having severe or critical disease. According to the guidelines set forth in the COVID‐19 Diagnosis and Treatment Plan (sixth edition) issued by China, patients meeting any of the following criteria are classified as having severe disease: (1) shortness of breath with a respiratory rate (RR) greater than or equal to 30 beats per minute; (2) resting oxygen saturation below 93%. Arterial partial pressure of oxygen (PaO_2_) to fraction of inspired oxygen (FiO_2_) ratio (PaO_2_/FiO_2_) ≤300 mmHg (1 mmHg = 0.133 kPa). Patients meeting any of the following conditions are classified as having critical disease: (1) Development of respiratory failure requiring mechanical ventilation, (2) onset of shock, and (3) presence of other organ failures necessitating ICU monitoring and treatment. The 74 patients involved in this study are severe or critically ill COVID‐19 patients, and were categorized into three groups based on treatment outcomes. *Perished cases*: The throat swab samples were collected three days before the patient died. SARS‐CoV‐2 virus nucleic acid is positive based on qRT‐PCR test of throat swab samples. *CDVC cases*: Even after 40 days of hospitalization, throat swab samples from the patients still tested positive for SARS‐CoV‐2 virus nucleic acid using qRT‐PCR. Although some clinical symptoms showed partial improvement, certain symptoms like fever and cough persisted. These cases, characterized by delayed clearance of the virus, were referred to as CDVC patients with prolonged viral presence. Additionally, the initial positive result for SARS‐CoV‐2 was detected within 3 days from the corresponding date. A total of 13 cases met these inclusion criteria and were included in our study. *Recovered case*s: Patients no longer had a fever for at least 3 days, respiratory symptoms were significantly improved or returned to normal, no dyspnea, percutaneous oxygen saturation (SpO_2_) ≥ 95% at a rest stage. CT chest imaging showed that the lung had lost the glabrous glass shape or was largely reabsorbed, no other organ displayed severe indication, and fully recovered and discharged within 7 days after the samples were collected. The SARS‐CoV‐2 nucleic acid was positive <30 days based on qRT‐PCR tests of pharyngeal swab samples, the viral nucleic acid was negative for more than 7 days when the samples were collected, and no subsequent positive tests.

### 2.3 Data collection

We reviewed clinical records, nursing records, laboratory findings (including complete blood count, serum biochemical test, coagulation profile, as well as serum cytokines), and chest x‐rays or CT scans for all the patients. All information was obtained and curated with a customized data collection form. Two investigators (Yi Zhang and Qinghua Qiao) independently reviewed the data collection forms to verify data accuracy.

### 2.4 Flow cytometric analysis

Peripheral blood samples from patients were harvested with anticoagulants (EDTA‐K_2_). Further experimental procedures were completed under biosafety level II plus condition. Flow cytometry analysis was conducted in seven separate batches, along with biochemical and cytokine analyses. Laboratory analysis of the samples was performed on the same day or within 1 day of the flow cytometry analysis. Please note that the results of the laboratory analysis for individual patient samples may not be available on the same day as the flow cytometry analysis. The flow loop gate strategy has been shown in Figure 3A. IL‐5, IFN‐ α, IL‐2, IL‐6, IL‐1 β, IL‐10, IFN‐ γ, IL‐8, IL‐17, IL‐4, IL‐12p70, and TNF‐ α were detected according to the instructions of the kit by using Reskedo heavy microsphere flow immunofluorescence photometer. A series of pre‐mixed multiple‐color, multiple‐monoclonal antibody reagent tubes were used to identify different cell subsets of whole blood specimens. The following tubes were used: DuraClone IM Phenotyping Basic Tube (B53309, Beckman Coulter), DuraClone IM T subsets Tube (B53328, Beckman Coulter), DuraClone IM B cells Tube (B53318, Beckman Coulter), DuraClone IM Treg Tube (B53346, Beckman Coulter). After being stained according to the standardized protocol, all samples were detected by a DxFLEX flow cytometry system (Beckman Coulter) and analyzed with the FlowJo software (TreeStar).

### 2.5 Statistical analysis

Statistical analyses were performed using GraphPad Prism version 8.0 (GraphPad Software, Inc.). Continuous variables were directly expressed as a range or median (interquartile range). Categorical variables were expressed as numbers/NUMBERS (%). *p* Values are from χ2 test, ordinary one‐way ANOVA, or Kruskal–Wallis test.

### 2.6 Study approval

This study was approved by the Ethics Committee of Wuhan Huoshenshan Hospital (approval document no.: HSSLL011), and all patients signed informed consent.

### 2.7 Role of the funding source

Funding agencies did not participate in study design, data collection, data analysis, or manuscript writing. The corresponding authors were responsible for all aspects of the study to ensure that issues related to the accuracy or integrity of any part of the work were properly investigated and resolved. The final version was approved by all authors.

## 3 RESULTS

### 3.1 Complications and clinical outcomes of severe COVID‐19

In this study, we retrospectively analyzed 74 cases of severe/critically ill COVID‐19 patients who received ICU admission in Huoshenshan Hospital in Wuhan, Hubei Province. These patients were 48 males and 26 females, with an average age of 63.95 years, and 36 patients were over 60 years old. Thirty‐eight patients had underlying metabolic diseases, including hypertension (*n* = 29, 39.19%), type‐I diabetes (*n* = 17, 22.97%), and cardiovascular disease (*n* = 9, 8.1%). All of these patients suffered from acute respiratory distress syndrome based on available arterial blood gas data. Less common complications included respiratory failure (*n* = 6, 8.11%), secondary infection (*n* = 2, 2.70%), acute cardiac injury (*n* = 8, 10.81%), acute kidney injury (*n* = 4, 5.40%) and acute liver injury (*n* = 3, 4.05%). The clinical symptoms included cough (*n* = 65, 87.84%), fever (*n* = 58, 78.38%), dyspnea (*n* = 50, 67.57%), fatigue (*n* = 46, 62.16%), myalgia (*n* = 21, 28.38%), and expectoration (*n* = 13, 17.57%). All of these severe COVID‐19 patients were given empirical antimicrobial treatment including moxifloxacin and/or cephalosporin, as well as antiviral therapy like oseltamivir and/or ganciclovir. Moreover, 28 (37.84%) cases received corticosteroid (methylprednisolone) during the course of hospitalization. Twenty‐four (32.43%) cases required noninvasive mechanical ventilation and 1 (1.35%) cases required invasive mechanical ventilation (Table 1). February 2020 marked the early stage of the COVID‐19 outbreak, during which limited large‐scale evidence‐based medicine was available. Therefore, the treatment plan for the patients in this study was established through collective discussions involving multiple departments and doctors. This approach can be categorized as empirical treatment, as it relies on the collective expertise and shared knowledge of the medical team rather than solely relying on established evidence‐based guidelines.

**Table 1:** Demographics and baseline characteristics of patients with severe COVID‐19.

	All patients		Perish		CDVC		Recovery		*p* Value
	(*N* = 74)		(*N* = 9)		(*N* = 13)		(*N* = 52）		
Female, *n*	26	35.1%	0	0%	4	30.8%	22	42.3%	0.046
Male, *n*	48	64.9%	9	100%	9	69.2%	30	57.7%	
Age, years	63.95	28–87	73	53–87	65.92	49–83	61.88	28–85	0.036
>60 years, *n*	36	48.6%	8	88.9%	9	69.2%	19	36.5%	<0.01
**Coexisting disorders**									
Any, *n*	43	58.1%	7	77.8%	6	46.2%	30	57.7%	0.33
Hypertention, *n*	29	39.2%	3	33.3%	4	30.8%	22	42.3%	0.69
Diabetes, *n*	17	23.0%	3	33.3%	3	23.1%	11	21.2%	0.72
Cardiovascular disease, *n*	9	8.1%	2	22.2%	2	15.4%	5	9.6%	0.52
Respiratory failure, *n*	6	8.1%	2	22.2%	2	15.4%	2	3.8%	0.10
Secondary infection, *n*	2	2.7%	2	22.2%	0	0%	0	0%	<0.01
Acute cardiac injury, *n*	8	10.8%	3	33.3%	2	15.4%	3	5.8%	0.041
Acute kidney injury, *n*	4	5.4%	3	33.3%	0	0%	1	1.9%	<0.01
Acute liver injury, *n*	3	4.1%	1	11.1%	1	7.7%	1	1.9%	0.33
**Symptoms**									
Fever, *n*	58	78.4%	7	77.8%	10	76.9%	41	78.8%	0.99
Maximum temperature, °C	38.06	36.2–40.0	38.08	36.2–39.8	38.36	36.3–40.0	37.98	36.5–39.8	0.40
Cough, *n*	65	87.8%	7	77.8%	13	100%	45	86.5%	0.25
Dyspnea, *n*	50	67.6%	8	88.9%	10	76.9%	32	61.5%	0.20
Fatigue, *n*	46	62.2%	6	66.7%	11	84.6%	29	55.8%	0.15
Myalgia, *n*	21	28.4%	2	22.2%	7	53.8%	12	23.1%	0.081
Expectoration, *n*	13	17.6%	1	11.1%	2	15.4%	10	19.2%	0.82
Headache, *n*	4	5.4%	1	11.1%	1	7.7%	2	3.8%	0.62
Coma, *n*	10	13.5%	9	100%	1	7.7%	0	0%	<0.01
Systolic pressure, mmHg	131	102–180	133	116–149	135	96–177	130	102–180	0.54
Heart rate, bpm	92	65–131	92	74–131	95	65–120	91	65–130	0.75
Respiratory rate, per min	22	16–37	24	20–37	23	18–34	21	16–32	0.035
**Therapy**									
Antiviral therapy, *n*	74	100%	9	100%	13	100%	52	100%	NA
Corticosteroid therapy, *n*	28	37.8%	9	100%	7	53.8%	12	23.1%	<0.01
Noninvasive mechanical ventilation, *n*	24	32.4%	8	88.9%	9	69.2%	7	13.5%	<0.01
SARS‐CoV‐2 IgM, AU/mL	38.7	10.6–163.5	48.8	15.0–83.4	55.5	6.7–163.5	30.3	0.6–80.3	0.018
>50 AU/mL, *n*	22	29.7%	4	44.4%	6	46.2%	12	23.1%	0.16
<50 AU/mL, *n*	52	70.3%	5	55.6%	7	53.8%	40	76.9%	
SARS‐CoV‐2 IgG, AU/mL	####	0.8–249.2	111.6	48.2–212.4	120.2	22.5–188.9	124.2	0.8–249.2	0.89
>100 AU/mL, *n*	37	50.0%	3	33.3%	7	53.8%	27	51.9%	0.56
<100 AU/mL, *n*	37	50.0%	6	66.7%	6	46.2%	25	48.1%	

After nearly 2 months of therapy, nine (12.16%) cases perished, with all of these cases being male, and the median number of days from illness onset to death being 47 days. Fortunately, 52 cases (70.27%) were categorized in the recovered group based on the definition provided above. Meanwhile, there were 13 (17.57%) cases with persistently positive qRT‐PCR tests of pharyngeal swab samples for SARS‐CoV‐2 after 40 days of hospitalization, even though the clinical symptoms in some patients were alleviated. These 13 patients were categorized as the CDVC group. CDVC cases included 9 males and 4 females, with an average age of 65.92 years, 9 cases were over 60 years old, and 6 cases had coexisting disorders, and none of them had secondary infection nor acute kidney injury. All cases received antiviral therapy, 7 (53.8%) cases had also received corticosteroid therapy, and 9 (69.2%) required noninvasive mechanical ventilation. Most importantly, 6 (46.2%) cases had high concentration of serum SARS‐CoV‐2 specific IgM antibody (>50 AU/mL) and 7 cases (53.8%) were positive for SARS‐CoV‐2 specific IgG antibody (>100 AU/mL), demonstrating that significant levels of both SARS‐CoV‐2 viral‐specific antibodies were found in some CDVC cases (Table 1).

### 3.2 Laboratory findings and computed tomography scans

**Figure 1:**
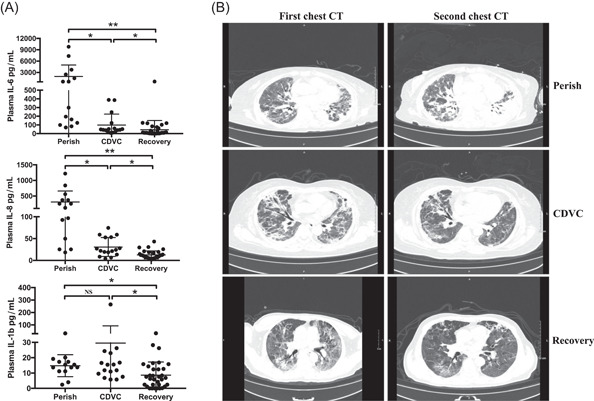
CDVC patients manifested high inflammation but chest CT showed obviously resolved ground‐glass opacity. (A) Serum concentrations of indicated cytokines in COVID‐19 patients. (B) Chest CT views of lung window from COVID‐19 patients. The first chest CT was tested in 7 days after hospitalization, and the second chest CT was performed about 14 days after the first test. In the perished group, one representative 67‐year‐old male showing severe bilateral ground‐glass opacity and consolidation by the first chest CT, progressive symptoms, and even presentation of “white lung” appearance in left lung by the second chest CT. In the CDVC group, a representative 54‐year‐old male showing bilateral ground‐glass opacity and partial consolidation by first chest CT, and second chest CT showing obviously resolved ground‐glass opacity. In the recovered group, a representative 62‐year‐old male showing moderate bilateral ground‐glass opacity by the first chest CT, and which has been resolved in the second chest CT. CT, computed tomography. *p < .05 and **p < .01.

High levels of pro‐inflammatory cytokines including IL‐2, IL‐6, IL‐8, and IL‐10 were observed in the serum of the perished group. Most importantly, the concentrations of IL‐6 (1928.2 pg/mL, range 71.2–9800 pg/mL) and IL‐8 (306.3 pg/mL, range 18.4–1220 pg/mL) were 357.1‐ and 14.9‐fold than the upper limit of normal range (ULN), respectively. Interestingly, serum concentrations of these cytokines were completely below ULN in the recovered group. However, in the CDVC group, although IL‐6 (98.2 pg/mL, range 8.7–388 pg/mL) and IL‐8 (30.7 pg/mL, range 4.2–74 pg/mL) were lower than those in the perished group, they were still higher than ULN. IL‐1β, a pro‐inflammatory factor that promotes tissue damage during viral infection,^10^, ^11^ was also increased in the CDVC group (25.8 pg/mL, range 0–264.7 pg/mL), and its level was significantly higher than the perished‐ and the recovered‐ groups, highlighting it may contribute to the clinical phenotypes observed (Figure 1A, Table 1). Serum concentrations of other cytokines, including IL‐5, IFN‐α, IL‐4, IL‐12p70, and TNF‐α, however, were not significantly different among the perished‐, CDVC‐, and recovered‐ groups (Table 2).

**Table 2:** Laboratory findings of patients with severe COVID‐19 after treatment.

		Perish (*n* = 9)			CDVC (*n* = 13)			Recovery (*n* = 52)			*p* Value*
	Normal range	Mean	Range	*n*	Mean	Range	*n*	Mean	Range	*n*	
**Cytokines tests**											
IL‐5	<3.1 pg/mL	3.5	2.1–6.8	14	3.2	1.9–7.2	16	2.7	1.6–5.2	33	0.055
IFN‐α	<8.5 pg/mL	2.7	1.9–3.7	14	3.4	0.7–22.0	16	2.8	0.6–18.2	33	0.26
IL‐2	<7.5 pg/mL	14.7	3.0–33.8	14	5.1	1.7–17.7	16	3.1	1.3–5.8	33	<0.01 b, c
IL‐6	<5.4 pg/mL	1928.2	71.2–9800	14	98.2	8.7–388	16	45.4	0.0–590	33	<0.01 a, b, c
IL‐1β	<12.4 pg/mL	14.8	2.4–32.2	14	29.6	5.8–264.7	16	8.7	0.0–32.8	33	<0.01 b, c
IL‐10	<12.9 pg/mL	22.6	2.7–114.5	14	6.9	2.5–21.5	16	4.2	2.2–13.5	33	<0.01 a, b
IFN‐γ	<23.1 pg/mL	17.3	2.0–80.8	14	7.4	1.6–16.9	16	4.3	0.2–12.1	33	<0.01 b, c
IL‐8	<20.6 pg/mL	306.3	18.4–1220	14	30.7	4.2–74	16	12.5	2.1–43	33	<0.01 a, b, c
IL‐17	<21.4 pg/mL	4.8	3.0–14.0	14	4.0	1.6–23.4	16	2.6	1.1–7.2	33	<0.01 a, b
IL‐4	<8.56 pg/mL	2.9	1.9–4.6	14	3.8	1.5–17.9	16	2.7	1.2–5.0	33	0.82
IL‐12p70	<3.4 pg/mL	2.7	2.1–3.2	14	4.5	1.6–18.8	16	2.8	1.5–10.1	33	0.14
TNF‐α	<16.5 pg/mL	3.2	0.0–7.6	14	4.8	0.3–23.9	16	3.5	0.3–10.8	33	0.90
**Laboratory tests**											
White blood cell count, WBC	3.5–9.5 × 10^9^/L	12.4	3.1–35.8	14	5.6	2.5–12.8	18	6.1	2.7–13.3	56	<0.01 a, b
Red blood cell count, RBC	4.0–5.8 × 10^12^/L	3.0	2.5–3.6	14	3.3	2.5–4.8	18	3.9	2.9–5.7	56	<0.01 b, c
Neutrophil ratio	40%–75%	89.1	72.9–96.0	14	71.2	52.1–96.6	18	56.8	41.3–76.8	56	<0.01 a, b, c
Neutrophil count	1.8–6.3 × 10^9^/L	11.2	2.3–34.3	14	4.1	1.7–12.4	18	3.5	1.4–10.2	56	<0.01 a, b
Lymphocyte ratio	20%–50%	6.2	2.4–24.4	14	18.5	0.8–34.5	18	30.6	13.3–48.1	56	<0.01 a, b, c
Lymphocyte count	1.1–3.2 × 10^9^/L	0.6	0.3–0.9	14	0.9	0.1–2.2	18	1.8	0.8–3.3	56	<0.01 b, c
Monocyte ratio	3%–10%	3.6	0.0–8.3	14	6.4	0.6–10.7	18	8.2	5.4–13.4	56	<0.01 a, b, c
Monocyte count	0.1–0.6 × 10^9^/L	0.4	0.0–1.0	14	0.3	0.0–0.8	18	0.5	0.2–1.1	56	<0.01 c
Hemoglobin, HGB	120–170 g/L	93.0	74.0–109.0	14	102.8	77.0–148.0	18	120.0	90.0–166.0	56	<0.01 b, c
Platelet count, PLT	125–350 × 10^9^/L	138.0	14.0–393.0	14	190.8	89.0–320.0	18	215.3	82.0–410.0	56	<0.01 b
C‐reactive protein, CRP	0–4 mg/L	109.1	15.8–242.9	14	38.2	0.8–254.5	17	4.4	0.0–50.3	55	<0.01 a, b, c
Fibrinogen, FIB	2–4 g/L	2.8	1.0–4.7	14	3.0	2.0–3.8	18	2.9	2.2–5.0	53	0.72
Activated partial thromboplastin time, APTT	21–37 s	38.3	24.2–68.4	14	31.1	23.3–37.4	18	28.3	21.7–38.8	53	<0.01 a, b
Prothrombin time, PT	9.2–15 s	16.1	11.7–26.0	14	13.6	11.8–16.0	18	12.8	10.7–17.3	53	<0.01 a, b
Thrombin time, TT	10–20 s	17.1	12.6–25.3	14	16.2	13.2–19.4	18	15.2	13.0–18.8	53	<0.01 b
d‐Dimer	0–0.55 mg/L	2.8	1.0–6.8	14	5.1	0.4–10.2	18	1.1	0.2–6.7	53	<0.01 b, c
Alanine aminotransferase, ALT	9–50 IU/L	45.4	8.6–320.5	14	24.7	8.5–91.4	18	40.7	4.4–521.6	56	0.59
Aspartate aminotransferase, AST	9–60 IU/L	40.0	14.9–102.0	14	22.7	12.1–46.3	18	27.3	12.2–232.9	56	0.17
Total protein, TP	65–85 g/L	54.5	40.4–63.3	14	61.9	45.0–79.5	18	66.3	53.6–88.5	56	<0.01 a, b, c
Albumin, ALB	40–55 g/L	31.5	26.7–41.6	14	35.2	24.5–43.8	18	38.1	32.2–47.1	56	<0.01 a, b, c
Globulin, GLB	20–40 g/L	23.0	13.3–27.8	14	26.0	14.6–40.6	16	28.7	19.1–46.7	44	<0.01 b
A/G	1–2.4	1.4	1.0–2.1	14	1.4	0.6–2.1	16	1.4	0.8–2.0	44	0.75
Total bilirubin, TBIL	0–26 µmol/L	67.3	5.8–554.5	14	11.9	4.9–27.7	18	9.2	4.0–19.8	56	<0.01 a, b
Direct bilirubin, DBIL	0–8 µmol/L	43.2	2.3–376.0	14	5.4	1.9–17.6	18	3.1	1.1–11.5	56	<0.01 a, b
Indirect bilirubin, IBIL	0–14 µmol/L	24.0	2.3–178.5	14	6.3	3.0–11.0	18	6.3	2.5–12.4	56	0.015 a, b
Total biliary acid, TBA	0–10 µmol/L	20.7	0.0–84.3	14	5.4	0.0–16.5	18	6.6	0.0–54.0	56	<0.01 a, b
Glucose, GLU	3.9–6.11 µmol/L	7.4	3.9–11.2	14	6.9	4.5–12.5	18	5.7	4.1–10.2	56	<0.01 b, c
Blood urea nitrogen, Urea	3.1–8 µmol/L	17.6	8.4–37.7	14	7.3	2.3–18.6	18	5.7	1.9–22.3	56	<0.01 b, c
Creatinine, Cre	57–97 µmol/L	78.4	42.1–232.9	14	74.5	46.0–203.7	18	65.6	32.0–124.5	56	0.22
Uric acid, UA	202–416 µmol/L	249.4	####–801.0	14	236.1	106.0–400.0	18	308.0	148.0–748.0	56	0.044
Alkaline phosphatase, ALP	45–125 IU/L	135.0	51.6–272.0	14	125.4	50.0–347.8	18	76.4	41.7–144.1	56	<0.01 b, c
Gamma glutamyltranspeptidase, GGT	10–60 IU/L	45.2	25.0–80.5	14	75.1	18.8–242.6	18	47.4	0.0–222.8	56	0.072
K	3.5–5.3 mmol/L	4.4	3.6–5.9	14	4.2	3.4–4.8	18	4.3	3.4–5.1	55	0.37
Na	137–147 mmol/L	148.3	####–165.1	14	142.9	133.6–158.4	18	141.2	134.7–146.4	55	<0.01 a, b
CL	99–110 mmol/L	109.3	95.3–130.1	14	105.7	98.6–116.0	18	106.0	93.9–112.3	55	0.056
Ca	2.11–2.52 mmol/L	2.2	2.0–2.6	14	2.2	1.8–2.5	18	2.3	2.0–2.5	55	<0.01 b
P	0.85–1.51 mmol/L	0.8	0.0–1.7	14	1.0	0.6–1.7	18	1.2	0.7–1.6	44	<0.01 b
Mg	0.75–1.02 mmol/L	1.0	0.8–1.5	14	0.9	0.7–1.1	18	0.9	0.8–1.1	44	0.018 a
Creatine kinase, CK	24–190 IU/L	71.4	13.2–275.3	14	38.5	11.3–112.6	17	44.0	18.2–192.8	56	0.043 a, b
Lactate dehydrogenase, LDH	120–250 IU/L	329.3	####–649.7	14	278.3	134.5–712.3	17	202.0	148.9–476.2	56	<0.01 b, c
a‐hydroxybutyrate dehydrogenase, HBHD	72–182 IU/L	270.1	####–593.5	14	237.1	98.6–645.6	17	157.4	114.2–332.0	56	<0.01 b, c
Creatine kinase‐MB, CK‐MB	0–24 IU/L	20.1	6.7–72.0	14	10.6	5.3–31.8	17	9.1	0.0–17.6	56	<0.01 a, b
Cystatin C, CysC	0.63–1.25 mg/L	1.8	1.1–2.7	14	1.3	0.9–2.6	18	1.1	0.7–1.7	56	<0.01 a, b, c
CO_2_	22–29 mmol/L	26.3	18.5–35.7	14	27.6	22.1–37.1	18	24.9	18.6–32.1	56	0.012 c
Procalcitonin, PCT	0–0.05 ng/mL	2.8	0.1–12.3	14	0.2	0.0–1.3	15	0.1	0.0–0.4	39	<0.01 a, b
Myoglobin, MYO	0–80 ng/mL	392.4	21.9–1913	11	22.1	3.4–84	9	7.5	1.1–29	27	0.016 a, b
Troponin, TNI	0–0.04 ng/mL	0.5	0.0–5.1	11	0.1	0.0–0.3	9	0.0	0.0–0.0	27	0.19
Natriuretic peptide type B, BNP	0–100 pg/mL	407.3	41.5–1816	14	947.5	0.0–1965	8	119.7	0.0–2157	27	0.001 c

* a: Perish versus CDVC *p* < .05; b: Perish versus Recovery *p* < .05; c: CDVC versus Recovery *p* < .05.

To further clarify the inflammation status of these patients, we next tested other inflammation‐associated biochemical indicators. Interestingly, most of perished cases manifested high levels of these factors, for example, enhancement of C‐reactive protein (CRP, 109.1 mg/L, range 15.8–242.9 mg/L), total bilirubin (TBIL, 67.3 μmol/L, range 5.8–554.5 μmol/L), direct bilirubin (DBIL, 43.2 μmol/L, range 2.3–376.0 μmol/L), indirect bilirubin (IBIL, 24.0 μmol/L, range 2.3–178.5 μmol/L), total biliary acid (TBA, 20.7 μmol/L, range 0–84.3 μmol/L), blood urea nitrogen (urea, 17.6 μmol/L, range 8.4–37.7 μmol/L), alkaline phosphatase (ALP, 135.0 IU/L, range 51.6–272.0 IU/L), lactate dehydrogenase (LDH, 329.3 IU/L, range 220.5–649.7 IU/L), α‐hydroxybutyrate dehydrogenase (HBHD, 270.1 IU/L, range 166.9–593.5 IU/L), procalcitonin (PCT, 2.8 ng/mL, range 0.1–12.3 ng/mL), myoglobin (MYO, 392.4 ng/mL, range 21.9–1913 ng/mL), and natriuretic peptide type B (BNP, 407.3 pg/mL, range 41.5–1816 pg/mL) was observed in perished group, suggesting an increased level of systemic inflammation (Table 2). However, all of these parameters were restored to normal range in the recovered group. On the other hand, in the CDVC patients, CRP (38.2 mg/L, range 0.8–254.5 mg/L), LDH (278.3 IU/L, range 134.5–712.3 IU/L), HBHD (237.1 IU/L, range 98.6–645.6 IU/L), and BNP (947.5 pg/mL, range 0–1965 pg/mL) were lower than those in the perished patients, but still higher than the normal range, demonstrating that systemic inflammation was still present in CDVC cases. In addition, other parameters have returned to the normal range (Table 2).

Interstitial lung abnormalities were observed in chest computed tomography (CT) scans of all patients on admission. The typical findings of chest CT images of severe COVID‐19 on admission showed bilateral ground‐glass opacity and subsegmental areas of consolidation, then progressed rapidly with mass shadows of high density in both lungs. Representative chest CT images of perished cases showed that the bilateral ground‐glass opacity was maintained. However, subsequent chest CT images revealed that the bilateral ground‐glass opacity had been resolved in CDVC and recovered cases (Figure 1B).

**Figure 2:**
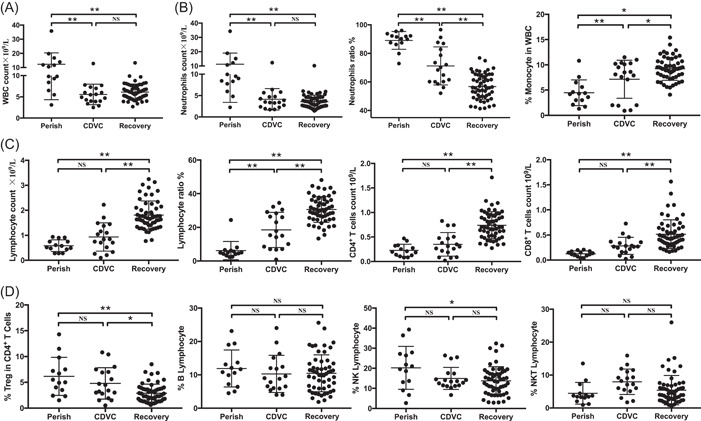
The count and frequency of immune cell subsets in severe/critically ill COVID‐19 patients after treatment. Statistical analysis of (A) the numbers of WBC; (B) the number and percentages of neutrophils and monocytes; (C) the count and percentages of lymphocytes; and (D) the frequency of other kinds of cells, including Treg, B cells, NK cells and NKT cells in perished, CDVC, and recovered patients. NS, not significantly different, *p < .05 and **p < .01.

The absolute numbers of white blood cells (WBC, 1.24 × 10^10^/L, range 3.1–35.8 × 10^9^/L) in the perished group were 1.31 folds above the ULN, and these patients also had high percentage (89.1%, range 72.9%–96%) and absolute number (1.12 × 10^10^/L, range 2.3–34.3 × 10^9^/L) of neutrophils. However, the numbers of both WBC and neutrophils in the CDVC and recovered cases returned to normal levels (Figure 2A,B and Table 2). Lymphopenia (lymphocyte count <0.8 × 10^9^/L) is the main feature of severe COVID‐19 patients.^8^, ^12^ Eleven (84.6%) perished cases had lymphopenia and the mean number of lymphocytes was 0.6 × 10^9^/L (range 0.3–0.9 × 10^9^/L). Mild recovery of lymphocyte counts was observed in CDVC patients but the absolute number (0.9 × 10^9^/L, 0.1–2.2 × 10^9^/L) was still below the lower limit of normal range (LLN) (Figure 2C and Table 2).

### 3.3 Enhancement of PD‐1 on effector T cells from CDVC

Immune cell subsets were further analyzed by flow cytometry, from which we observed that the frequency and absolute numbers of CD4^+^ (39%, range 10.9%–57.1%; 0.23 × 10^9^/L, range 0.08–0.47 × 10^9^/L), and CD8^+^ T cells (21.1%, range 10.3%–26.3%; 0.13 × 10^9^/L, range 0.03–0.21 × 10^9^/L) in perished cases were significantly lower than LLN, whereas, the frequency and absolute numbers of these cells had returned to normal levels in recovered group. Enhancement of frequency and counts of CD4^+^ T cells (35.4%, range 19.5%–53.4%; 0.35 × 10^9^/L, range 0.07–0.83 × 10^9^/L), and CD8^+^ T cells (31.6%, range 14.3%–48.8%; 0.28 × 10^9^/L, range 0.02–0.73 × 10^9^/L) were also seen in CDVC cases, but the levels were still lower than recovered group (Figure 2C, Table 3). CD25^+^Foxp3^+^CD4^+^ regulatory T cells (Tregs) have an important inhibitory effect during viral infection.^13^, ^14^, ^15^ Interestingly, the frequencies of Tregs in perished (6.2%, range 1.5%–14.3%) and CDVC (4.8%, range 0.5%–10.8%) groups were higher than those in recovered cases (2.9%, range 0.6%–8.5%; Figure 2D, Table 3). We also analyzed the percentage and total number of B cells, NK cells, and NKT cells within these patients, and results showed that no significant difference in the proportions of these cell types was observed (Figure 2D, Table 3).

**Table 3:** T cell features of patients with severe COVID‐19.

	Perish (*n* = 9)		CDVC (*n* = 13)		Recovery (*n* = 52)		*p* Value*
	Mean	Range	Mean	Range	Mean	Range	
**CD4** ^**+**^ **T cell subsets**							
% CD4^+^ in lymphocyte	###	10.9–57.1	###	19.5–53.4	###	11.4–64.6	0.09
CD4^+^ T cells count (10^9^/L)	###	0.08–0.47	###	0.07–0.83	###	0.27–1.7	<0.01 b, c
% Effector in CD4^+^ T cells	0.7	0.04–2.32	1.3	0.08–4.9	0.5	0.01–3.4	0.034 c
% Naïve in CD4^+^ T cells	###	9.2–48.8	###	4.1–28.7	###	4.9–54.6	0.039 c
% TCM in CD4^+^ T cells	###	31.0–66.9	###	26.6–76.6	###	23.2–82.6	<0.01 b
% TEM in CD4^+^ T cells	###	8.2–47.7	###	4.3–53.1	###	2.0–58.0	<0.01 b, c
% PD‐1^+^ in CD4^+^ T cells	###	6.5–29.2	###	9.1–41.6	9.6	1.1–31.6	<0.01 b, c
% PD‐1^+^ in CD4^+^ TCM cells	###	9.3–28.0	###	9.5–41.0	###	1.7–26.1	<0.01 b, c
% PD‐1^+^ in CD4^+^ TEM cells	###	12.3–62.7	###	9.0–77.0	###	1.1–63.8	<0.01 b, c
% PD‐1^+^ in CD4^+^ effector T cells	###	0–67.5	9.4	0–45.1	5.4	0–47.3	<0.01 a, b
**CD8** ^**+**^ **T cell subsets**							
% CD8^+^ in lymphocyte	###	10.3–26.3	###	14.3–48.8	###	9.5–51.6	0.034 a, b
CD8^+^ T cells count (10^9^/L)	###	0.03–0.21	###	0.02–0.73	###	0.16–1.56	<0.01 b, c
% Effector in CD8^+^ T cells	###	12–45.9	###	9.5–62.7	###	4.9–54.2	<0.01 b, c
% Naïve in CD8^+^ T cells	7.7	1.3–19.1	7.6	1.6–19.6	###	1.1–44.5	<0.01 b, c
% TCM in CD8^+^ T cells	7.4	1.9–13.4	7.7	1.6–16.5	###	1.7–38.7	0.011 b, c
% TEM in CD8^+^ T cells	###	37.9–81.5	###	31.2–68.3	###	19.4–76.8	0.25
% PD‐1^+^ in CD8^+^ T cells	###	11.4–61.9	###	7.4–69.3	###	2.1–28.9	<0.01 b, c
% PD‐1^+^ in CD8^+^ TCM cells	###	10–85.3	###	20.9–77.0	###	3.36–63.8	<0.01 b, c
% PD‐1^+^ in CD8^+^ TEM cells	###	9.6–77.7	###	11.0–77.9	###	0.8–38.4	<0.01 b, c
% PD‐1^+^ in CD8^+^ effector T cells	###	2.9–38.6	###	0.8–56.9	2.8	0.1–16.3	<0.01 b, c
**CD25** ^**+**^ **Foxp3** ^**+**^ **Treg cell subsets**							
% Treg in CD4^+^ T cells	6.2	1.5–14.3	4.8	0.5–10.8	2.9	0.6–8.5	<0.01 b, c
Treg cells count (10^9^/L)	0.012	0.005–0.041	0.012	0.003–0.028	0.020	0.004–0.071	<0.01 b, c
% Naïve Treg in Treg cells	9.6	2.7–17.0	8.6	1.3–17.2	###	0.8–39.9	<0.01 c
% Memory Treg in Treg cells	###	77.6–97.3	###	68.2–98.7	###	59.7–99.2	0.15
% Active Treg in Treg cells	###	10.1–83.5	###	12.9–70.1	###	8.6–80.7	0.20
**Other cell subsets**							
T Lymphocyte cells count (10^9^/L)	###	0.16–0.68	###	0.05–1.67	###	0.48–2.75	<0.01 b, c
% B Lymphocyte in Lymphocyte Cells	###	4.5–23.1	###	4.0–24.0	###	1.9–25.6	0.99
B Lymphocyte cells count (10^9^/L)	0.086	0.02–0.37	0.084	0.02–0.21	0.196	0.02–0.51	<0.01 b, c
% NK lymphocyte in lymphocyte cells	###	2.7–39.3	###	6.7–26.4	###	2.9–32.4	0.028 b
NK lymphocyte cells count (10^9^/L)	###	0.01–0.25	###	0.01–0.43	###	0.05–0.87	<0.01 b, c
% NKT lymphocyte in lymphocyte cells	4.4	0.9–13.5	7.9	1.7–15.9	5.4	0.8–26	0.39
NKT lymphocyte cells count (10^9^/L)	0.026	0.005–0.07	0.073	0.006–0.23	0.091	0.013–0.33	0.04 b

* a: Perish versus CDVC *p* < .05; b: Perish versus Recovery *p* < .05; c: CDVC versus Recovery *p* < .05.

**Figure 3:**
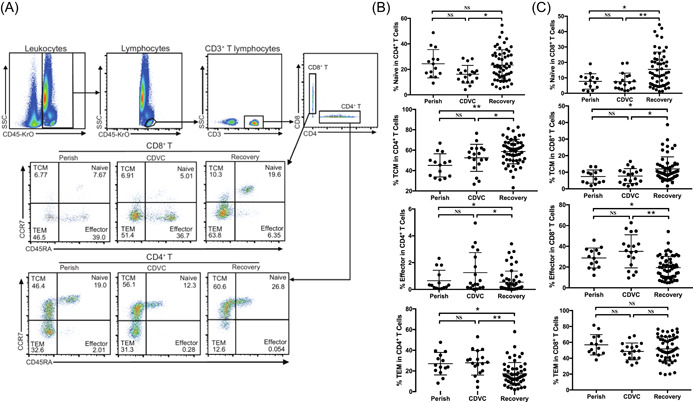
The status of T cells from severe/critically ill COVID‐19 patients after treatment. (A) Representative CCR7 and CD45RA expression on circulating T cells from one CDVC case was measured by flow cytometry. (B) Statistical analysis of indicated cells in perished, CDVC, and recovered patients. NS, not significantly different, *p < .05 and **p < .01.

We next examined the functional status of T cells in peripheral blood. T cells were classed into four groups based on CCR7 and CD45RA expression, and flow cytometry showed that the recovered patients had higher percentage of CCR7^+^CD45RA^+^ naïve CD4^+^ and CD8^+^ T cells in circulation than perished and CDVC groups (Figure 3, Table 3). The naïve T cells can differentiate into CCR7^+^CD45RA^−^ central memory T cells (TCM); therefore, the recovered patients also manifested higher frequency of CD4^+^ and CD8^+^ TCM than perished as well as CDVC cases (Figure 3, Table 3). On the other hand, CDVC and perished patients had higher percentage of CCR7^−^CD45RA^+^ effector CD4^+^ and CD8^+^ T cells than recovered group (Figure 3, Table 3), suggesting that the persistence of SARS‐CoV‐2 would trigger T cell activation in these patients. After activation, most of the effector T cells are apoptotic, and the rest differentiate into memory T cells. Flow cytometry also confirmed that both perished and CDVC cases have higher percentage of CCR7^−^CD45RA^−^ effector memory T cells (TEM) than recovered group (Figure 3, Table 3).

**Figure 4:**
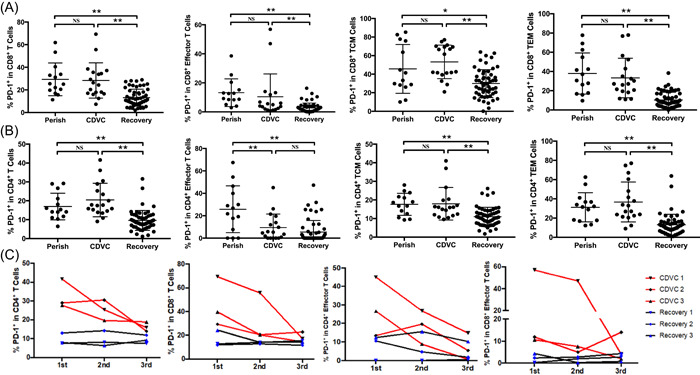
Augmenting PD‐1 on T cells from CDVC patients. Statistical analysis of (A) PD‐1 on CD8^+^ T cells; (B) PD‐1 on CD4^+^ T cells in perished, CDVC, and recovered patients. (C) Time‐dependent expression of PD‐1 on indicated T cells from CDVC (*n* = 3) and recovered patients (*n* = 3). The first flow cytometry was tested 7 days after hospitalization, and the second and the third time was performed 7 and 14 days after the first test, respectively. NS, not significantly different, *p < .05 and **p < .01.

Our previous work found that T cells upregulated expression of the exhaustion marker PD‐1 in severe COVID‐19 patients, suggesting that SARS‐CoV‐2 induces T cell exhaustion during infection.^8^ We therefore analyzed the expression of PD‐1 on circulating T cells. Surprisingly, both perished and CDVC cases manifested significantly higher frequencies of PD‐1 on CD4^+^ and CD8^+^ T cells than the recovered group. Most importantly, the frequency of PD‐1 on CD8^+^ effector, CD8^+^ TCM, and CD8^+^TEM cells was significantly higher than in recovered cases (Figure 4A, Table 3). Additionally, the frequency of PD‐1 on CD4^+^ TCM and CD4^+^ TEM cells was also dramatically higher than those in the recovered group (Figure 4B, Table 3). Finally, time‐dependent decreasing PD‐1 on circulating CD4^+^, CD8^+^ T cells, and effector T cells was also observed in CDVC cases after treatment, whereas, the expression of PD‐1 on T cells was not changed dramatically in the recovered group following inpatient care (Figure 4C). Collectively, these data indicate these effector T cells were functionally exhausted in CDVC, leading to ineffective clearance of the SARS‐CoV‐2 virus.

## 4 DISCUSSION

Recently, many studies, including ours, have also reported that SARS‐CoV‐2 infection can trigger immune system damage, especially in severe cases. Actually, previous work showed that COVID‐19 patients are categorized into several groups based on clinical features.^16^ Nevertheless, no clinical and immunological data are available regarding COVID‐19 patients who received medical treatment. In February of 2020, the Chinese government successfully set up two temporary hospitals (Huoshenshan and Leishenshan) in Wuhan to cure severe/critically ill COVID‐19 patients. After two months of treatment, while a small amount of these severe patients died, an overwhelming percentage of cases have recovered. Unfortunately, some of them still carried SARS‐CoV‐2 during the hospitalization, a phenomenon called delayed virus clearance. We here retrospectively reviewed the clinical features of 13 CDVC cases from Huoshenshan Hospital. The results showed that CDVC patients still had high serum concentrations of IL‐6, IL‐1β, IL‐8, and pro‐inflammatory indicators, including CRP, LDH, HBHD, and BNP, but lower than those in the perished group. Moreover, these patients also manifested severe lymphopenia, as the counts of total lymphocytes, CD4^+^ T cells, and CD8^+^ T cells were extremely low compared to those in the recovered group. Most importantly, CDVC cases had higher levels of PD‐1 on effector, TCM, and TEM CD4^+^ and CD8^+^ T cells, leading to T cell exhaustion, and consequent inefficiency of SARS‐CoV‐2 clearance. To our knowledge, it is the first report on the clinical and immunological features of CDVC cases.

The mechanisms underlying the severe pathogenicity of COVID‐19 are not fully understood, although extensive lung damage in SARS‐CoV‐2 infected patients appears to be associated with increased monocyte, macrophage, and neutrophil infiltration in the lungs.^17^, ^18^ Moreover, elevated levels of serum pro‐inflammatory cytokines and chemokines were also observed in severe/critically ill COVID‐19 cases.^8^, ^9^, ^12^ Therefore, the clinical deterioration of SARS‐CoV‐2 infection may result from a combination of direct virus‐induced cytopathic effects and immunopathology induced by cytokine storm. Cytokine storm is a phenomenon of excessive inflammatory reaction in which high levels of cytokines are rapidly produced against microbial infection, which has been considered an important contributor to immune‐pathological damage during SARS‐CoV and MERS‐CoV infection.^19^ High levels of pro‐inflammatory cytokines (IL‐2, IL‐6, IL‐7, TNF‐α, G‐CSF, MCP‐1, and MIP‐1A) were also found in severe COVID‐19 patients compared to individuals with uncomplicated SARS‐CoV‐2.^4^ Consistent with this report, we here also found that serum from the perished group manifested high levels of IL‐2, IL‐6, and IL‐8 and IL‐10. Interestingly, all of these cytokines are restored to normal range in recovered cases. However, CDVC patients still have high levels of serum IL‐6, IL‐8, and IL‐1β, demonstrating that these patients still have inflammation in vivo. Type I IFN (IFN‐I) forms part of the very early immune response to viral infections.^20^ It has been shown that SARS‐CoV infections result in a delayed expression of IFN‐I, which was found to promote the accumulation of pathogenic inflammatory monocyte/macrophages, resulting in elevated lung cytokine levels, vascular leakage, and impaired virus‐specific T‐cell responses.^21^ Nevertheless, we here found that serum from perished and CDVC patients had very low levels of IFN‐α (Table 2), suggesting that IFN‐I might not contribute to the immune‐pathological processes involved in lung injury during SARS‐CoV‐2 infection.

Lymphopenia is another clinical presentation of immune damage caused by SARS‐CoV, MERS‐CoV, and SARS‐CoV‐2 infection. SARS‐CoV has been shown to significantly decrease CD4^+^ and CD8^+^ T cell counts,^22^, ^23^ and MERS‐CoV has been shown to efficiently infect T cells to cause T cell apoptosis leading to lymphopenia.^24^ Similar to our previous work, we also found that both perished and CDVC cases showed severe lymphopenia, while a rescue of lymphocyte counts was observed in the recovered group (Figure 2, Table 2). At present, little is known about the mechanism underlying the lymphopenia caused by SARS‐CoV‐2 infection. However, one possibility is that the lymphopenia is mediated by pro‐inflammatory cytokines. After all, both IL‐6 and IL‐1β accumulated in serum from perished and CDVC cases (Table 2), can mediate pyroptosis, a pro‐inflammatory form of cell apoptosis.^25^ We also reported that the serum IL‐6 was negatively correlated to peripheral blood CD4^+^ and CD8^+^ T cell counts in COVID‐19 patients.^8^ It is worth noting that the patients who died during the 1997 H5N1 influenza outbreak also showed lymphoid depletion associated with a high titer of circulating cytokines, including IL‐6.^26^, ^27^ Our recent work demonstrated that SARS‐CoV‐2 cannot directly infect human T or B cells; whereas, it infects macrophages and DCs in lymph nodes to trigger IL‐6 and IL‐1β secretion, suggesting that macrophages and DCs‐derived IL‐6 and IL‐1β can trigger lymphocyte apoptosis and necrosis.^16^ Finally, we could not exclude the possibility that some of the lymphopenia may be worse due to the use of steroids during hospitalization, further research is required to determine the effects of corticosteroids on lymphocytes in the context of COVID‐19.

In a mouse SARS‐CoV model, it seems that the depletion of CD4^+^ T cells at the time of infection leads to enhanced immune‐mediated interstitial pneumonitis and delayed viral clearance.^28^ Conversely, CD8^+^ T cells infiltrating into the pulmonary interstitium play a critical role in mediating virus clearance in SARS‐CoV‐infected patients.^22^, ^29^ Moreover, T cells are able to kill virus‐infected cells in the MERS‐CoV‐infected mice,^30^ highlighting the importance of T cells in controlling SARS‐CoV and MERS‐CoV infection. We here also found that the perished and CDVC patients have high percentage of effector CD4^+^ and CD8^+^ T cells due to the persistent of SARS‐CoV‐2 in vivo (Figure 3, Table 3), suggesting that SARS‐CoV‐2 viruses induce T cell activation. Nevertheless, persistent viral antigen stimulation may also induce T cell exhaustion, a state of T cell dysfunction that manifests loss of cytokine production capability and reduced functions. By flow cytometry analysis, we found that both CD8^+^ T cells and CD4^+^ T cells have higher levels of exhaustion marker PD‐1 in CDVC patients (Figure 4), reflecting the immune dysfunction observed in CDVC patients, as the immune response is abnormally skewed towards immunosuppressive Th2 response. Most importantly, the frequency of PD‐1 on CD8+ effector, CD8+ TCM, CD8+ TEM, CD4+ TCM, and CD4+ TEM cells in CDVC and perished cases was significantly higher than that in the recovered group. IL‐10, an inhibitory cytokine, not only prevents T cell proliferation, but also can induce T cell exhaustion.^31^ Important, blocking IL‐10 function has been shown to successfully prevent T cell exhaustion in animal models of chronic infection.^32^, ^33^ We demonstrate here that some perished and CDVC patients have very high levels of serum IL‐10 (Table 2), suggesting that IL‐10 might be mechanistically responsible for T‐cell exhaustion. Collectively, these data indicate these effector T cells were functionally exhausted in CDVC, leading to ineffective clearance of the SARS‐CoV‐2 virus.

T cells play an essential role in fighting against viral infection. Therefore, there is an urgent need to find novel strategies to boost the number and function of T cells in patients. Tocilizumab, a humanized anti‐IL‐6 receptor antibody, has been approved for the treatment of severe COVID‐19 patients, and whether tocilizumab can restore T cell numbers in COVID‐19 patients is an interesting investigation. On the other hand, Thymosin‐α1 (Tα1), a polypeptide hormone produced by thymic epithelial cells, can effectively increase T cell numbers, support T cell differentiation, maturation, and reduce cell apoptosis.^34^, ^35^ Therefore, Tα1 has been successfully used in clinical practice to treat patients infected with hepatitis B (HBV), hepatitis C (HCV), and human immunodeficiency viruses (HIV), and its efficacy has been demonstrated by pathological observation.^36^, ^37^, ^38^ Currently, our group has shown that Tα1 effectively enhances T cell counts in COVID‐19 patients with severe lymphopenia. Because most of CDVC patients have lymphocytopenia, our results may support them to apply Tα1 injection to improve immune function.

Our study has some limitations. First, the data regarding the viremia profile of SARS‐CoV‐2 are not available, and further studies are needed to investigate the correlation between the viral load kinetics and the dynamics of cellular immune response. Secondly, only throat swab samples were collected from all enrolled participants, and there was a lack of lower respiratory tract samples for immunological analysis. We lack information on the current form of the pandemics, such as changes in treatment or the emergence of new varieties. A recent report revealed that 22 of the 133 patients admitted with COVID‐19 had an initial or follow‐up positive sputum or fecal sample after the conversion of their pharyngeal samples from positive to negative.^39^ We are uncertain as to whether or not the 52 cases in the recovered group in our study had SARS‐CoV‐2 in additional body sites and its influences on immunological response. Systematic and simultaneous collection of specimens from multiple body sites during future evaluations may be necessary to clarify this potentially confounding factor. Finally, some CDVC cases have higher and more sustainable serum levels of SARS‐CoV‐2 specific IgM and IgG antibodies, despite simultaneously carryed SARS‐CoV‐2, suggesting that antibody responses may not be sufficient to determine virus clearance in these patients. Gaining a deeper understanding of the factors that affect antibody responses and their association with SARS‐CoV‐2 in the host is important for the clinical management of CDVC patients.

In conclusion, the CDVC group represents a distinct subset of patients with specific characteristics. These findings emphasize the importance for physicians to thoroughly investigate and manage these individuals. It is crucial to consider that prolonged positivity for the virus may have implications for decisions regarding isolation programs and appropriate patient care.

## AUTHOR CONTRIBUTIONS

Yuzhang Wu, Yi Zhang, and Qinghua Qiao designed the experiments. Qinghua Qiao, Xuemin Sun, Wenjiong Shi, Hao Li, and Zhenhua Zhang contributed to patient recruitment, experiments, and data collection. Jinsong Wang, Debao Li, and Bo Tang analyzed the data. Yi Zhang and Qinghua Qiao interpreted the results and wrote the article.

## CONFLICT OF INTEREST STATEMENT

The authors declare no conflict of interest.

## ETHICS STATEMENT

This study was approved by the National Health Commission of China and the Ethics Commission of Huoshenshan Hospital.

## Data Availability

The data that support the findings of this study are available from the corresponding author upon reasonable request.
